# Comparative Evaluation of Fat Quality in Conventional and Specialist Infant Formulas

**DOI:** 10.3390/molecules30153221

**Published:** 2025-07-31

**Authors:** Aleksandra Purkiewicz, Joanna Browarek, Renata Pietrzak-Fiećko

**Affiliations:** 1 Department of Commodity Science and Food Analysis, Faculty of Food Science, University of Warmia and Mazury in Olsztyn, Plac Cieszyński 1, 10-718 Olsztyn, Poland; aleksandra.purkiewicz@uwm.edu.pl; 2 Sensory Laboratory of the Institute of Animal Reproduction and Food Research of the Polish Academy of Sciences in Olsztyn, Trylińskiego 18, 10-683 Olsztyn, Poland; j.browarek@pan.olsztyn.pl

**Keywords:** fat quality, food for infants, fatty acid profile

## Abstract

This study assesses the quality of fat in conventional and specialist infant formulas (IFs) available in Poland. The IFs studied were characterized in terms of fatty acid profiles and lipid quality indices. The study material consisted of eight types of conventional and specialist IFs. The determination of fatty acids was carried out using gas chromatography (GC). Lipid quality indices were estimated based on established formulas. Goat milk-based formulas showed significantly higher levels of caproic acid (C6:0) and capric acid (C10:0) than cow milk-based formulas of the same category (initial or follow-on) (*p* < 0.05). In addition, these IFs stood out in terms of conjugated linoleic acid (CLA) content (0.30%) compared to cow and specialist formulas (about 0.20%). It was shown that the average ratio of *n*6/*n*3 fatty acids was significantly lower in conventional IFs (6.07:1) compared to specialist IFs (8.10:1). The goat’s milk-based IFs had the most favorable values for individual lipid quality indices (index of desirable fatty acids (DFAs) = 62.46; index of hypercholesterolemic fatty acids (OFAs) = 25.94; index of atherogenicity (AI) = 0.71; index of thrombogenicity (TI) = 0.88; hypocholesterolemic/hypercholesterolemic ratio (H/H) = 2.05), while the specialist S-PH formula was characterized by the lowest DFA value (49.17) and the highest AI and TI indices (1.48 and 1.68). Multivariate analysis clearly classified the division of formulas into two groups—conventional and specialist—based on lipid quality indices. The results obtained provide new information on the variation in the lipid profile of IFs depending on the intended use of the formula and may serve as a basis for further research in this area.

## 1. Introduction

Breastfeeding is the most recommended method of infant nutrition, demonstrating multifaceted health benefits for both child and mother [1]. Human milk (HM) is optimized in composition and adapts individually to the needs of the infant, covering the need for all essential nutrients and shaping the development of the young organism [2]. When there are contraindications to breastfeeding, including maternal infection with HIV, HTLV, cytomegalovirus, and hepatitis C, it is necessary to supplement or replace HM with infant formula (IF) [3].

The use of IFs is becoming increasingly widespread. According to global statistics, between 2015 and 2020, only approximately 44% of infants aged 0–6 months were exclusively breastfed, highlighting the growing reliance on formula feeding [4]. IFs have been developed as a complete alternative to HM, which are able to meet the full nutritional needs of infants under 12 months of age [5]. The classification of IFs by age of infants includes three categories—first-feeding (initial) IFs (0–6 months), follow-on feeding IFs (6–12 months), and IFs for young children (over 1 year of age) [6]. Depending on the child’s health status and special nutritional, digestive, or metabolic needs, IFs are divided into conventional and specialist. Conventional IFs are intended for healthy infants without specific nutritional needs, in cases where breastfeeding is not possible for various reasons. These IFs are mostly based on cow’s milk, and less frequently on goat’s milk. In contrast, specialist IFs are used in the nutrition of infants with dietary requirements, such as food allergies or intolerances, metabolic disorders, digestive issues, or in cases of prematurity [7]. The production of specialist IFs requires the use of advanced processing techniques, including enzymatic hydrolysis and high-pressure techniques, as well as strict control of quality parameters and adjustment of composition to meet the specific nutritional needs of infants [8,9].

Lipids in IFs can contribute up to 55% of their total energy value. They not only provide energy for the infant but also play a crucial role in the proper development of the nervous system, growth, and the absorption of fat-soluble vitamins [10,11]. The fatty acid composition of IFs is directly influenced by the type of lipid sources used in their formulation. For their production, vegetable fats such as palm oil, canola oil, sunflower oil, coconut oil, or soybean oil, as well as milk fat in some cases, are most used [10,12]. The addition of palm oil results in a high palmitic acid (C16:0) content, while the addition of coconut oil increases the content of short- and medium-chain fatty acids [13]. Although IFs are designed to closely mimic the composition of HM, current technological limitations and differences in raw materials make it impossible to fully replicate the lipid profile of HM. As a result, significant differences exist not only in the proportions of individual fatty acids but also in their positional distribution within triglycerides, which affects fat digestibility and metabolism in infants [14].

The type of milk used in production can have a significant impact on the lipid composition of IFs. Goat milk is richer than cow’s milk in short-chain fatty acids (SCFAs) [15]. It is indicated that selected trans fatty acids (TFAs), including conjugated linoleic acid (CLA), are found in higher amounts in goat milk [16]. These differences directly affect the composition of IFs by differentiating them in terms of fatty acid composition. In addition, the lipid profile of IFs differs depending on their intended use. Conventional IFs, intended for healthy infants, are based on a blend of vegetable oils (mainly sunflower oil and canola oil), fish oil, algae oil, and milk fat [17]. In contrast, specialist IFs, including protein hydrolysates or amino acid formulas, are composed of blends of vegetable oils, with the additional inclusion of palm oil, palm olein, and coconut oil. The different sources of fat in the two types of formulas can affect the proportions of the different classes of fatty acids and the ratios of each other’s fatty acids, especially from the polyunsaturated fatty acid group.

Lipid quality in food products is typically determined through a comprehensive analysis of the fatty acid composition and the relationships among individual fatty acids or their groups. Several indices are used to characterize lipid quality, such as the desirable fatty acid (DFA) index, the hypocholesterolemic/hypercholesterolemic (H/H) ratio, and the atherogenicity (AI) and thrombogenicity (TI) indexes [18]. Despite growing interest in the quality of lipids in IFs [3,10,19], few studies have been published to date comparing the detailed fatty acid composition of conventional and specialist IFs with specific nutritional needs. To our knowledge, this is the first study conducted in Poland to include both conventional and specialist IFs. Importantly, previous work has rarely included analyses of lipid quality indices, including AI, TI, or DFAs, which can be a valuable tool for assessing the pro- or anti-inflammatory and metabolic potential of these products.

In the present study, a comparative assessment of the fatty acid profiles and lipid quality indices was conducted for selected conventional and specialist IFs available on the Polish market. The aim of the analysis was to compare the content of individual fatty acid fractions between these groups of products and to evaluate their nutritional value using lipid quality indices, such as the AI, TI, H/H, DFAs, and OFAs.

## 2. Results

### 2.1. Composition and Types of IFs Used in This Study

Table 1 presents the IFs used in this study. The research material included four conventional IFs intended for initial and follow-on feeding, as well as four specialist IFs. The table provides a simplified composition of the formulas, obtained from the manufacturers’ labels, along with the nutritional purpose and specific characteristics of each product. These data served as background information for interpreting the observed differences in fatty acid content and ratios among the analyzed formulas. Details regarding sample selection and the criteria for choosing the types of IFs are described in Section 4.2.

In this study, the fatty acid profiles of the analyzed IFs were compared based on relative values (expressed as a percentage of the total fatty acid content). This approach enables a comparison of the lipid profile structure among the formulas, regardless of their total fat content. Although the label data indicated some differences in the declared total fat content (ranging from 22.2 to 26.4 g), these values were not used as a basis for quantitative analysis in this work. Therefore, the results presented in this study were interpreted in the context of the proportions of fatty acids within the lipid mixture of the IFs rather than their absolute amounts in the final product.

### 2.2. Fatty Acid Profiles of the Tested IFs

Table 2 shows the content of individual fatty acids and the sums of the selected fatty acid groups in the conventional and specialist IFs studied. A total of thirty-seven fatty acids were identified, including fourteen saturated fatty acids (SFAs), five monounsaturated fatty acids (MUFAs), six polyunsaturated fatty acids (PUFAs)—three n-6 and three n-3, five branched-chain fatty acids (BCFAs), and seven trans fatty acids (TFAs). In all IFs, the predominant fatty acids were palmitic acid (C16:0) (15.63–28.67%), oleic acid (C18:1 *n*9) (27.14–41.15%), and linoleic acid (C18:2 *n*6) (10.23–16.92%).

Of the SFAs, two acids from the SCFA group were identified in the IFs analyzed—butyric acid (C4:0) and caproic acid (C6:0). Of the specialist IFs, only S-PH contained the indicated acids. The content of medium-chain fatty acids (MCFAs)—caprylic (C8:0), capric (C10:0), and lauric (C12:0)—ranged from 7.23 to 11.14% in conventional IFs and from 8.53 to 11.74% in specialist IFs. The K-CM2 and S-PH formulas stood out in terms of SFA content, as determined by higher myristic (C14:0), pentadecylic (C15:0), and palmitic (C16:0) acid content (*p* < 0.05), while K-GM2 stood out in terms of MUFAs, with a significant predominance of oleic (C18:1 *n*9) acid (*p* < 0.05). Both conventional IFs for follow-on infant feeding (K-CM2 and K-GM2) contained higher amounts of selected SCFAs (butyric, C4:0) and MCFAs (caprylic and capric, C8:0 and C10:0) than the corresponding IFs for initial infant feeding (K-CM1 and K-GM1). The goat milk-based IFs (K-GM1 and K-GM2) contained lower palmitoleic (C16:1 *n*7) acid contents relative to the other IFs (*p* < 0.05). In the context of PUFA, the specialist formula S-PH contained the lowest levels of LA (linoleic acid), ALA (α—linolenic acid), and DHA (docosahexaenoic acid) (*p* < 0.05) and did not contain EPA (eicosapentaenoic acid), while also exhibiting the lowest total PUFA content. In the specialist IFs, the total PUFA content varied over a wide range—from 12.28 to 19.50%. Specialist IFs were characterized by lower fatty acid diversity, such as the lack of branched-chain fatty acids (BCFAs) in S-S, S-FH, and S-A, and lower TFA, including the absence of CLA in three IFs (S-S, S-FH, S-A). Among TFAs, vaccenic acid (VA) was predominant in the analyzed IFs, ranging from 1.20% to 1.93%. It accounted for nearly 70% of total TFAs in conventional IFs and over 90% in specialist formulas.

PUFA *n*6/PUFA *n*3 ratios ranged from 5.50 ± 0.27 in K-GM1 to 8.90 ± 0.29 in S-PH. The average PUFA *n*6/PUFA *n*3 ratio in the specialist formulas was two units higher than in the conventional ones (8.10 vs. 6.07). The ratios of PUFAs varied depending on the type of formula. Higher DHA:AA (docosahexaenoic acid:arachidonic acid) ratios were observed in conventional IFs (ranging from 0.98:1 to 1.74:1) compared to specialized IFs (0.72:1 to 0.95:1). An opposite trend was noted for the LA:ALA ratio, with conventional formulas ranging from 6.66:1 to 8.79:1, while specialist formulas exhibited higher values, ranging from 9.25:1 to 11.13:1.

### 2.3. Evaluation of PUFA Compliance in Infant Formulas

Table 3 summarizes the total PUFA and individual PUFA (LA, ALA, AA, DHA, EPA) percentages determined by the manufacturer of IFs (VL) and the percentages of these acids determined experimentally (VM). The indicated percentages are given for seven IFs, due to the lack of complete information on the label of the S-FH formula, which makes it impossible to perform the necessary calculations. In most cases, the VMs of the total and individual PUFA types were consistent with the VL values reported by the manufacturers. The VMs of total PUFA content ranged from 95% to 111% of the VLs. For LA, the compliance between the VM and the VL ranged from 92% to 113%, and for ALA, from 85% to 118%. Deviations exceeding 20% from the declared values were observed in selected formulas for LCPUFAs. VMs were shown to be 20% or higher relative to VL for the S-PH and S-S IFs. The first had twice the AA content and more than 30% lower DHA content, while the second IFs had about 30% lower DHA content.

### 2.4. Evaluation of Lipid Quality Indices

Based on the fatty acid composition of the IFs, the values of selected lipid quality indices were estimated: DFA, OFA, AI, TI, and H/H (Table 4). The values of the DFA and OFA indices exhibited considerable variability. For conventional formulas, DFA ranged from 54.34 to 64.67, while OFA ranged from 23.01 to 36.04. In specialist formulas, these values were 49.17 to 61.08 for DFA and 34.93 to 41.37 for OFA, respectively. The K-GM1 and K-GM2 formulas exhibited the highest DFA and the lowest OFA values. Moreover, the K-GM2 formula, in addition to exhibiting the highest DFA and the lowest OFA values, also showed the lowest AI and TI indices and the highest H/H ratio, making it the most favorable formula in terms of lipid quality indices among those analyzed. The S-PH formula was characterized by the highest AI and TI indices (*p* < 0.05). A wide variation in the H/H index was observed, ranging from 0.93 in S-PH to 2.38 in K-GM2.

### 2.5. Grouping of IFs Based on the Lipid Quality Indices and Their Interrelationships

#### 2.5.1. Hierarchical Cluster Analysis (HCA)

**Figure 1:**
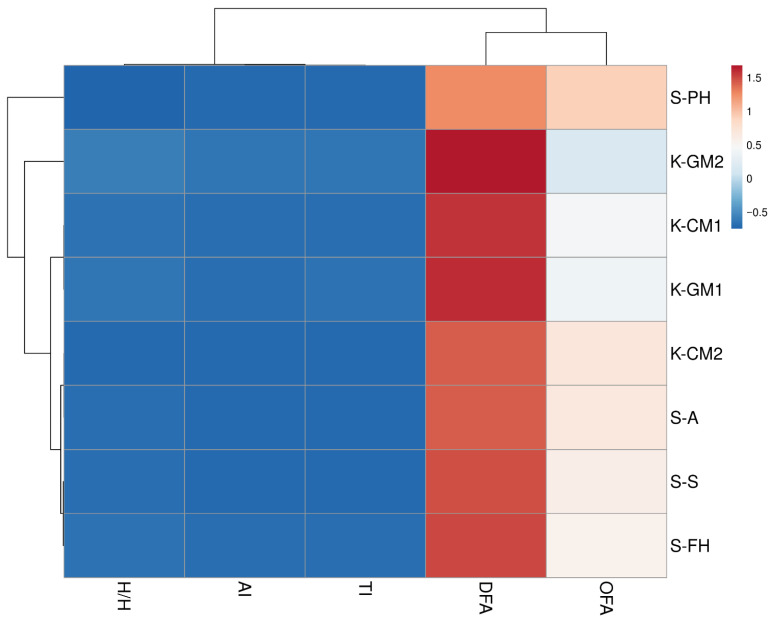
Clustered heat map with a dendrogram illustrating the lipid quality indices in IFs tested. Abbreviations: DFAs—index of desirable fatty acids, OFAs—index of hypercholesterolemic fatty acids, AI—index of atherogenicity, TI—index of thrombogenicity, H/H hypocholesterolemic/hypercholesterolemic ratio.

To illustrate the differences in lipid quality between the IFs analyzed, a hierarchical cluster analysis (HCA) was performed based on five lipid quality indices: DFA, OFA, AI, TI, and H/H (Figure 1). These indices consider both the quantitative and qualitative composition of fatty acids and provide a recognized tool for assessing the health-promoting potential of fat in foods. The data were previously rescaled using Z-score (unit variance scaling) standardization, which allowed a comparison of samples regardless of their absolute content of individual lipid fractions. Two-way hierarchical clustering (both rows and columns) was then performed using Euclidean distance and the average linkage method. The resulting matrix was visualized as a heat map with a dendrogram.

HCA enabled the identification of three main clusters of samples that differed in terms of lipid quality indices, while reflecting the division between conventional (K) and specialist (S) IFs. The first cluster included the S-PH formula, which showed the most different lipid profile compared to the other IFs, exhibiting the highest OFA, AI, and TI values and the lowest DFA values (*p* < 0.05). The second cluster included conventional formulas—K-GM2, K-GM1, and K-CM1—which had similar DFA and relatively low OFA values. In addition, the goat’s milk-based formula was characterized by the lowest AI index values among those analyzed, and the K-GM2 formula additionally had the highest H/H ratio. The third cluster included specialist formulas S-A, S-S, and S-PH and conventional formulas K-CM2, which also had moderately high values of the DFA, AI, TI, and H/H indices at similar levels to those of the formulas in cluster one.

#### 2.5.2. Correlation Analysis

**Figure 2:**
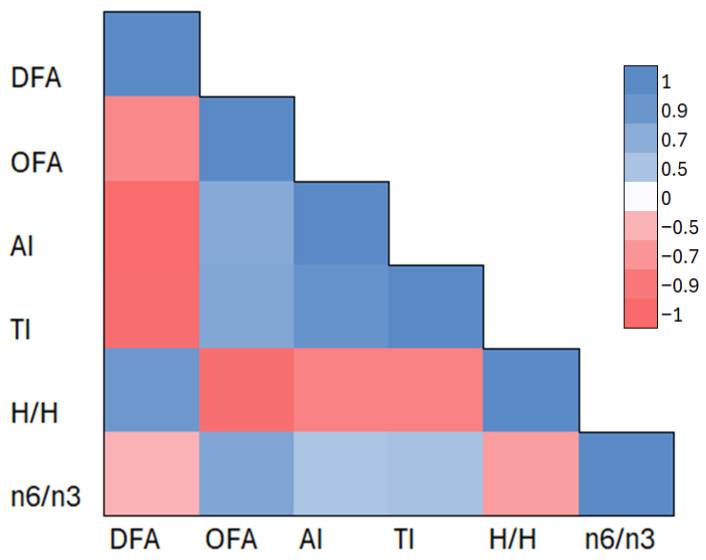
Correlation matrix of the selected lipid parameters in the IFs tested. Abbreviations: DFAs—index of desirable fatty acids, OFAs—index of hypercholesterolemic fatty acids, AI—index of atherogenicity, TI—index of thrombogenicity, H/H hypocholesterolemic/hypercholesterolemic ratio. The color scale represents the strength and direction of Pearson correlation coefficients (r), with red indicating negative correlations and blue indicating positive correlations. The analysis includes the following lipid quality indices: DFA, OFA, AI, TI, H/H, and the *n*6/*n*3 ratio.

Figure 2 shows the correlation matrix presented as a heat map between the analyzed lipid quality indices in the IFs tested. The coloring reflects the strength and direction of the correlation coefficient (r), with shades of red indicating negative correlations and blue indicating positive correlations. The DFA index was strongly negatively correlated with AI (r = −0.98), TI (r = −0.97), and OFA (r = −0.79). In contrast, the OFA index, defined as the sum of saturated fatty acids (lauric, myristic, palmitic acids), was strongly positively correlated with AI (r = 0.73) and TI (r = 0.76).

In addition, the *n*6/*n*3 ratio, representing the ratio of omega-*6* to omega-*3* fatty acids, showed slightly weaker correlations—it was positively correlated with AI (r = 0.50) and TI (r = 0.53) and negatively correlated with DFA (r = −0.50) and H/H (r = −0.64).

The correlation results confirm the opposing nature of lipid fractions considered favorable (DFA) and unfavorable (OFA) and their unambiguous effect on complex metabolic indicators such as AI, TI, and H/H. The correlation matrix reinforces the interpretation presented earlier based on the cluster analysis and heat map, highlighting the consistency of lipid patterns in the IFs studied.

## 3. Discussion

This study indicates that nutritional use and the composition of IFs can differentiate the fatty acid profiles of IFs. The higher content of selected SFAs—caproic and capric (C6:0 and C10:0) in goat milk-based formulas was due to the higher content of these acids in goat milk relative to cow’s milk [20]. Prosser et al. [21] showed that goat milk-based formula was richer in selected SFAs, including caproic and caprylic. Caproic, caprylic, and capric (C6:0, C8:0, and C10:0) acids are typical of goat milk and can account for up to 18% of the total fatty acid content of goat milk, compared to cow’s milk, where the content of these acids does not exceed 9% [22]. Acids from the SCFA and MCFA groups are more easily released and absorbed in the infant’s gastrointestinal tract, which contributes to the higher digestibility of goat milk fat compared to cow’s milk fat and classifies goat formulas as better digested by infants. For this reason, goat’s milk is recommended for consumers who are allergic to cow’s milk [22]. It has also been indicated that cow’s milk contains higher levels of palmitic acid (C16:0) [23], but no statistical differences were noted in the study material analyzed.

In the analyzed conventional IFs, the MUFA content was higher in goat milk-based formulas, which was primarily determined by the higher levels of oleic (C18:1 *n*9) and eicosenoic (C20:1 *n*9) acids. On the other hand, the available literature does not indicate significant differences in MUFA content between cow and goat milk-based IFs [21]. Given the limited number of comparative studies on the fatty acid profiles of cow and goat milk-based IFs, the observed differences in MUFA content may result from both the natural compositional differences between the two types of milk and the use of vegetable oils in the formulations, which are sources of MUFAs, particularly oleic acid. In the analysis conducted, nervonic acid (C24:1 *n*9)—despite its important role in the development of the infant central nervous system [24]—was not detected in any of the tested IFs. The absence of this compound may be attributed to the lack of clear regulatory requirements mandating its inclusion in IFs, both within the European Union and in other regions [25]. Although nervonic acid is naturally present in HM, its occurrence in IFs is not common. Some authors report trace amounts of this fatty acid in selected commercial IFs [26], while others did not identify it at all [10]. Yu et al. [26] found that among 118 tested IF samples, fewer than 54% contained nervonic acid. Moreover, the authors noted that its concentration in IFs was less than 16% of that found in human colostrum. The variability in nervonic acid content in IFs may result from differences in the analytical methodologies used to determine fatty acid profiles, as well as variations in the composition of raw materials employed by manufacturers. The primary fat sources in IFs—such as bovine/goat milk fat and vegetable oils—do not contain nervonic acid. Potential sources include oils derived from *Malania oleifera* and *Lunaria annua*, but due to limited research and the high content of erucic acid, these oils are not commonly added to IFs [26]. Given the significance of nervonic acid in neuronal myelination and cognitive development, its potential role in infant nutrition warrants further investigation and a critical assessment of the need for its supplementation in infant formula products.

The European Commission Regulation [27] provides guidelines for the content of individual fatty acids. The regulation indicates that TFA content should not exceed 3%, while in the IFs tested, the values of these acids ranged from 1.38 to 2.48%. A higher proportion of TFAs were found in conventional IFs compared to specialist ones. The higher content of TFAs in conventional formulas is associated with their natural presence in both cow’s and goat’s milk. The highest TFA levels were detected in goat formulas, which was primarily due to a significantly higher proportion of VA and CLA. Djordjević et al. [16] report that goat milk may contain higher levels of CLA compared to cow milk, which is attributed to differences in farming practices and a more diverse diet in goats.

In the case of PUFAs, both cow’s milk and goat’s milk, compared to HM, contain significantly lower amounts of acids from this group. For this reason, when composing IFs, manufacturers are obliged to follow certain ranges in the content of individual PUFAs. According to the standards, from 2020, DHA in IFs should be added in the amount of 20–50 mg/100 kcal, which is about 0.33–1% of all fatty acids. The S-PH formula contained 0.05% lower DHA content compared to the required lower level of this acid in IFs. The AA content should not exceed 1%, which is in line with the studies conducted [27,28,29]. On the other hand, another criterion regarding AA is that it should be added in an amount at least equal to that of DHA [30], which was not met in the K-CM2 formula, where the contents of AA and DHA were 0.31% and 0.54%. In turn, the determined DHA content in the S-S formula was more than 20% lower than the value declared by the manufacturer. The periodic monitoring of the composition of IFs and the verification of manufacturers’ compliance with established compositional standards are essential. However, in the present study, the verification of DHA and AA levels in the analyzed IFs should be considered a priority, including the analysis of multiple product batches to confirm the observed deviations from the established standards. An adequate addition of LCPUFAs to IFs is essential to ensure the appropriate intake of these fatty acids by infants during a period of rapid growth and development. DHA and AA are essential for the normal development of the nervous system, retina, and cognitive function [31]. Although these acids can be synthesized endogenously from ALA and LA, the ability of infants to synthesize them is limited due to incompletely developed enzymatic processes [10].

The ratio of ALA to LA is important in the context of infant nutrition for the optimal synthesis of long-chain polyunsaturated fatty acids (LCPUFAs)—DHA or AA. According to the guidelines, the recommended LA:ALA ratio should range from 5:1 to 15:1 [32], which corresponds to a proportional contribution of LA and ALA at the levels of 12–15% and 1.5–2.5%, respectively. In the studies conducted, the LA:ALA ratio ranged from about 6:1 in the goat formulas to 11:1 in the extensively hydrolyzed protein formulas. According to the literature, ratios of these acids closer to the 5:1 value appear to promote higher endogenous synthesis of DHA [32]. Higher LA:ALA ratios observed in specialist IFs resulted from a distinct lipid composition, characterized by using vegetable oils rich in LA (e.g., sunflower or soybean oil) and a limited contribution of ALA sources, such as rapeseed or linseed oil. It should be noted that in more than 30% of countries worldwide, the LA:ALA ratio exceeds the recommended ratio of 15:1 [32].

There is a limited number of studies in the literature addressing lipid quality indices in IFs. Lipid quality in food products is assessed through a detailed analysis of the fatty acid profiles and the relative proportions of selected fatty acids. Desirable fatty acid (DFA) content is defined as the sum of MUFA, PUFA, and stearic acid (C18:0) [18]. The lowest DFA in the S-PH formula was due to the lowest MUFA and PUFA content relative to the other IFs. The highest DFA levels were recorded in goat milk-based IFs (K-GM1, K-GM2), which were enriched with fish oil and *Mortierella alpina* oil, which are sources of DHA and AA acids [33]. Moreover, high levels of DFA were observed in specialist infant formulas—those based on the soy protein (S-S) and extensively hydrolyzed protein (S-FH)—which was determined by the presence of oils derived from *Schizochytrium sp.* microalgae [34]. It has been reported that, compared to other fatty acids, lauric and myristic (C12:0 and C14:0) exert a cholesterol-raising effect, contributing to increased total cholesterol levels [35]. The high level of OFA in S-PH was due to the significantly high concentration of both myristic and palmitic (C14:0 and C16:0), determined primarily by the high proportion of palm oil and palm olein in their composition. Despite the rather wide range of OFA index values (23.01–41.37), the sum of lauric and myristic (C12:0 and C14:0) in the tested IFs is appropriate and does not exceed the established regulatory value of 20%. The AI and TI indices consider the potential influence of individual fatty acids on the development of atherosclerotic lesions (AIs) and the risk of thrombus formation (TI). The AI index describes the relationship between major SFAs and MUFAs and PUFAs, while the TI index examines the relationship between acids in the prothrombotic group (selected SFAs) and antithrombotic acids (MUFAs and PUFAs) [18]. Lower values of both ratios are more beneficial for cardiovascular health. The S-PH formula had the highest index value (AI = 1.48), which was associated with a significantly higher content of lauric, myristic, and palmitic (C12:0, C14:0, and C16:0) acids (41.37%), compared to the other IFs (23.01–37.70%), and statistically the lowest PUFA concentration. The same formula had the highest TI value (TI = 1.68), similarly due to the highest SFA content—myristic, palmitic, and stearic (C14:0, C16:0, and C18:0) acids (41.58%)—compared to the other IFs (26.22–35.78%), and lower MUFA and PUFA content. Despite the standardization of IFs and the existence of defined compositional norms that manufacturers are obliged to follow, the use of different proportions of vegetable oils in IFs affects the final concentrations of SFAs, MUFAs, and PUFAs, thereby influencing the values of the analyzed lipid quality indices. In the context of IFs, manufacturers are not required to report or declare AI and TI values; instead, the key requirement is compliance with the fatty acid composition criteria specified in Commission Delegated Regulation (EU) 2016/128 [27], which establishes the minimum and maximum permissible levels of total fat and selected fatty acids. The last indicator analyzed was H/H, which determines the relationship between hypocholesterolemic fatty acids (oleic (C18:1 *n*9), PUFA) and hypercholesterolemic fatty acids (lauric, myristic, palmitic; C12:0, C14:0, C16:0) [18]. The goat K-GM2 formula had the highest H/H ratio, indicating a higher proportion of hypocholesterolemic fatty acids, with higher oleic content for this formula (*p* < 0.05).

The results of the correlation analysis indicated a strongly negative relationship between the DFA index and AI, TI, and OFA, confirming that higher levels of MUFAs and PUFAs in the preparations translated into lower OFA values and lower atherogenic and thrombogenic potential. This relationship is well illustrated by the goat K-GM2 formula, characterized by the highest DFA values and, at the same time, the lowest AI and TI values. In contrast, the S-PH formula characterized by the highest OFA values also showed the highest AI and TI values. The specialist formulas studied had significantly higher *n*6/*n*3 acid ratios compared to conventional IFs. These formulas had a higher content of omega 6 acids relative to omega 3 acids (ALA, DHA, EPA). This suggests that formulas with a higher *n*6/*n*3 ratio may contain a lower proportion of *n*3 PUFA. Such a trend was noted in the S-PH formula, which, while having the highest *n*6/*n*3 ratio (8.90 ± 0.29), also had the lowest content of omega 3 acids (1.24%). It should be noted that AI and TI increase with increasing SFAs (lauric, myristic, palmitic acids; C12:0, C14:0, C16:0), and decrease with increasing PUFAs [18,36]. Specialist formulas often contain vegetable oils rich in LA (soybean oil, sunflower oil, corn oil), and include coconut oil and palm oil, rich in lauric (C12:0), myristic (C14:0) and palmitic (C16:0) acids, which may explain their less favorable lipid profile.

### Strengths and Limitations of This Study

The strengths of the present study include conducting a comparative analysis of conventional and specialist IFs in terms of lipid fraction profiles. In addition, relatively few studies so far have included an analysis of lipid quality indices in assessing the fat quality of IFs. This study may fill a gap in the literature regarding the assessment of lipid quality in IFs. However, the limited availability of the literature on this topic hindered a full comparative evaluation and limited the depth of result interpretation. It is necessary to conduct further analyses covering a wider range of IFs, both conventional and specialist, and to include more batches of the same products. Such efforts will allow confirmation of the observed trends and more representative conclusions. The study presented should be regarded as a preliminary study, providing a starting point for further, more comprehensive comparative analyses. In future studies, in addition to including more IFs in the study material, the authors also plan to consider including sheep’s or mare’s milk-based formulas to observe other correlations.

## 4. Materials and Methods

### 4.1. Chemicals

Ammonia, potassium hydroxide, and sodium sulfate were purchased from Sigma Chemical Co. (St. Louis, MO, USA). Methyl and ethyl alcohols, as well as diethyl ether and petroleum ether, were obtained from Merck (Darmstadt, Germany).

### 4.2. Sample Selection

The study material consisted of selected IFs purchased in commercial stores and pharmacies in Olsztyn (Poland). All the IFs studied were in powder form and packaged in a cardboard box or aluminum can. The selection of IFs for this study was based on their stationary availability in the city of Olsztyn. This study included conventional IFs for initial (for infants up to 6 months of age) and follow-on (after 6 months of age) feeding of infants based on cow’s and goat’s milk, as well as specialist IFs for feeding infants with special nutritional needs. Eight IFs were analyzed, including four conventional (for initial and follow-on infant feeding) and four specialist IFs. The selected conventional IFs were intended for healthy infants and nutritionally complete until 6 months of age (first-feeding IFs) or consumed after 6 months of age (follow-on IFs) with appropriate complementary foods, based on cow’s milk (K-CM1, K-CM2) or goat’s milk (K-GM1, K-GM2). Of the specialist IFs, the study material included partial hydrolyzed formula dedicated to infants with gastrointestinal problems (S-PH), extensively hydrolyzed formula for infants diagnosed with milk protein allergy (S-FH), soy formula consumed in lactose intolerance and during the need to eliminate cow’s milk protein from the diet (S-S), and amino acid formula consumed in severe forms of milk protein food allergy (S-A) [37]. The detailed composition, nutritional purpose, and descriptions of the tested formulas can be found in the Section 2.1 in Table 1.

### 4.3. Fat Extraction

IF samples were prepared by dissolving the appropriate amount of powder in water, following the manufacturer’s instructions as outlined in Table 1. Fat was extracted using the Rose–Gottlieb method [38]. For each sample, approximately 10 g (±0.01 g) of reconstituted formula was weighed, then mixed sequentially with 2 mL of a 10% ammonia solution and 10 mL of ethanol, with gentle mixing after each addition. Subsequently, 25 mL of a mixture of diethyl ether and petroleum ether was used to extract the fat. The resulting upper organic phase was separated and passed through anhydrous sodium sulfate to remove moisture. Solvents were then evaporated using a rotary evaporator. To enhance extraction efficiency, the procedure was repeated twice.

### 4.4. Chromatographic Determination of Fatty Acids

All types of fatty acids were converted into the corresponding fatty acid methyl esters (FAMEs) according to the standard procedure developed by the International Dairy Federation (IDF, 2002) [39], using a methanolic solution of potassium hydroxide (KOH). The methyl esters obtained were analyzed using gas chromatography (GC). The separation was carried out on a Hewlett–Packard 6890 gas chromatograph (Palo Alto, CA, USA) equipped with a flame ionization detector (FID) and a Supelcowax 10 capillary column (Supelco, Sigma-Aldrich, St. Louis, MO, USA; 100 m in length, 0.25 mm inner diameter, 0.25 µm film thickness, Supelcowax 10 stationary phase). Analysis conditions included the following temperatures: detector—250 °C, dispenser—230 °C, column—195 °C. Helium was used as carrier gas, with a flow rate of 1.5 mL/min (51 cm/s), split 50:1. The initial oven temperature was set at 50 °C and held for 1 min. Then, the temperature increased at a rate of 25 °C/min to 200 °C, followed by 3 °C/min to 230 °C, and held at 230 °C for 18 min. The identification of FAMEs was carried out by comparing retention times with values obtained for a standard mixture of 37 fatty acid methyl esters (Supelco 37 Component FAME Mix, 10 mg/mL in dichloromethane). The identification of individual BCFAs was performed by comparing their retention times and peak profiles with those of reference standards (*iso* C13:0, *iso* C14:0, *iso* C15:0, *anteiso* C15:0, *iso* C16:0) (Larodan Fine Chemicals, Sweden). Fatty acid composition was quantified using ChemStation (G1701BA B.01.00; Agilent, Alpharetta, GA, USA). Results were presented as a weight percentage relative to the total FAME content [40]. Figure S1 illustrates representative chromatograms used for compound identification.

### 4.5. Lipid Quality Indices

Lipid quality indices were determined using Equations (1)–(5), as described by Paszczyk and Tońska [18] and Pietrzak-Fiećko and Kamelska-Sadowska [36].

Index of Desirable Fatty Acids (DFAs)

(1) DFA=UFA+C18:0

UFAs—unsaturated fatty acids (MUFA + PUFA)

Index of Hypercholesterolemic Fatty Acids (OFAs)

(2) OFA=C12:0+C14:0+C16:0

Index of Atherogenicity (AI)

(3) $$AI=\frac{\left(\left(C12:0+4*C14:0\right)+C16:0\right)}{\left(PUFA\;n3+PUFA\;n6+MUFA\right)}$$

Index of Thrombogenicity (TI)

(4) $$TI=\frac{\left(C14:0+C16:0+C18:0\right)}{\left(\left(\left(0.5*C18:1\;n9\right)+0.5*sum\;of\;other\;MUFA\right)+0.5*PUFA\;n6+\left(3*PUFA\;n3\right)+\left(\frac{PUFA\;n3}{PUFA\;n6}\right)\right)}$$

Hypocholesterolemic/Hypercholesterolemic Ratio (H/H)

(5) $$\frac{H}{H}=\frac{\left(C18:1\;n9+C18:2\;n6+c18:3\;n3\right)}{\left(C12:0+C14:0+C16:0\right)}$$

### 4.6. Statistical Analysis

Data were expressed as mean values ± standard deviation (SD). The normality of variable distribution in the samples was assessed using the Shapiro–Wilk test, while Levene’s test was applied to verify the homogeneity of variances. Differences in fatty acid content and lipid quality indices among the IFs were evaluated using one-way analysis of variance (ANOVA) followed by Tukey’s post hoc test. Relationships between specific lipid quality indices were examined using Pearson’s correlation coefficient. To explore the variation in lipid quality based on these indices, hierarchical cluster analysis (HCA) was performed. A significance level of *p* < 0.05 was considered statistically relevant. Statistical analyses were carried out using Microsoft Excel (Microsoft 365, Redmond, WA, USA) and Statistica 13.1 (StatSoft Inc., Tulsa, OK, USA). Heat map visualization of the HCA results was generated using the ClustVis visualizing tool.

## 5. Conclusions

This study provides new information on the lipid quality of conventional and specialist IFs, with particular emphasis on differences in fatty acid composition and related lipid quality indices. Conventional IFs based on goat milk contained higher amounts of caproic acid (C6:0) and capric acid (C10:0) than cow milk-based formulas within the same category (initial or follow-on) (*p* < 0.05). In addition, conventional formulas were more diverse in terms of TFA and BCFA content as compared to specialist formulas. IFs based on goat’s milk were more abundant in CLA than those based on cow’s milk (0.31% vs. 0.20%). Specialist IFs had a higher LA:ALA ratio than conventional IFs (9.25:1–11.13:1 vs. 6.66:1–8.79:1), which was determined by oils rich in omega 6 acids in the ingredients. Conventional goat milk-based IFs were characterized by the most favorable lipid quality indices, with the highest DFA and H/H values and the lowest OFA, AI, and TI. Hierarchical cluster analysis clearly classified the division of formulas into two groups—conventional and specialist—based on lipid quality indices. Although this study addresses an important aspect, due to the limited number of samples analyzed, further analysis is recommended, including a larger number of IFs and the consideration of different batches of products. The extended analysis will allow a more complete assessment of lipid quality in IFs and the formulation of clear conclusions and nutritional and regulatory recommendations.

## Figures and Tables

**Table 1:** Characteristics of infant formulas used in this study.

No	Code	Use *	Description of the Product *	Nutritional Value *	Preparation *
1	K-CM1	First-feeding IFs.	Conventional based on cow’s milk	E 478 kcal, F 24.6 g (of which SFA 11.6 g, MUFA 8 g, PUFA 3.6 g), C 52.9 g (of which S 52.2 g), P 9.4 g	13.8 g of powder + 90 mL of water
2	K-GM1	First-feeding IFs.	Conventional based on goat’s milk, contains DHA	E 511 kcal, F 26.4 g (of which SFA 9.3 g, MUFA 12.4 g, PUFA 4.1 g), C 57.4 g (of which S 57.4 g), P 10.1 g	12.9 g of powder + 90 mL of water
3	K-CM2	Follow-on feeding IFs.	Conventional based on cow’s milk	E: 472 kcal, F 22.2 g (of which SFA 10.4 g, MUFA 7.6 g, PUFA 3.5 g), C 56.2 g (of which S 55.6 g), P 9.7 g	14.4 g of powder + 90 mL of water
4	K-GM2	Follow-on feeding IFs.	Conventional based on goat’s milk, contains DHA and AA	E: 511 kcal, F 26.4 g (of which SFA 9.3 g, MUFA 12.4 g, PUFA 4.1 g), C 57.4 g (of which S 57.4 g), P 10.1 g	12.9 g of powder + 90 mL of water
5	S-PH	Dietary management of gastrointestinal disorders, infant colic, and constipation. Nutritionally complete for infants up to 6 months of age.	Partially hydrolyzes milk proteins for easier digestion and faster gastric emptying. Contains B-palmitate and galacto-oligosaccharides	E: 496 kcal, F 25.9 g (of which SFA 13.3 g, MUFA 8.9 g, PUFA 3 g), C 51.1 g (of which S 5.9 g), P 12.6 g	13.5 g of powder + 90 mL of water
6	S-S	Dietary management of cow’s milk protein intolerance, lactose intolerance, galactosemia, and cow’s milk protein allergy. Nutritionally complete for infants up to 6 months of age.	Based on soy proteins	E: 489 kcal, F 23.4 g (of which SFA 9.9 g, MUFA 9.2 g, PUFA 4.3 g), C 58.2 g (of which S 5 g), P 11.3 g	14.1 g of powder + 90 mL of water
7	S-FH	Dietary management of cow’s milk protein allergy and other food allergies (such as soy protein), lactose intolerance, and secondary sucrose intolerance. Intended for the next feeding of infants.	Hypoallergenic, based on casein hydrolysate with a high degree of hydrolysis, contains probiotics	E: 504 kcal, F 25.2 g (of which SFA 10.4 g, MUFA nd g, PUFA nd g), C 57 g (of which S 25.9 g), P 12.4 g	13.5 g of powder + 90 mL of water
8	S-A	Dietary management of infants from birth to 12 months with cow’s milk protein allergy, eosinophilic esophagitis, and other indications where amino acid preparations are recommended.	Hypoallergenic, suitable as the only food source for infants under 12 months, contains LCPUFA, probiotics, and prebiotics	E: 472 kcal, F 23.6 g (of which SFA 10.4 g, MUFA 9 g, PUFA 3.8 g), C 50 g (of which S 4.7 g), P 13.2 g	14.4 g of powder + 90 mL of water

Abbreviations: * the information was obtained from the manufacturer’s original packaging label; E—energy value, F—fat, SFAs—saturated fatty acids, MUFAs—monounsaturated fatty acids, PUFAs—polyunsaturated fatty acids, C—carbohydrates, S—sugars, P—protein, nd—no data.

**Table 2:** Fatty acid profiles of conventional and specialist IFs tested, %.

Fatty Acids	Common Name	K-CM1	K-CM2	K-GM1	K-GM2	S-PH	S-S	S-FH	S-A
Saturated fatty acids (SFAs)									
C4:0	Butyric	1.12 ± 0.04 ^c^	1.43 ± 0.01 ^b^	0.94 ± 0.01 ^d^	1.16 ± 0.02 ^c^	1.57 ± 0.00 ^a^	ND	ND	ND
C6:0	Caproic	0.82 ± 0.04 ^c^	1.04 ± 0.01 ^b^	1.02 ± 0.05 ^b^	1.22 ± 0.02 ^a^	1.05 ± 0.03 ^b^	ND	ND	ND
C8:0	Caprylic	0.98 ± 0.07 ^d^	1.41 ± 0.07 ^b^	1.17 ± 0.03 ^c^	1.51 ± 0.03 ^b^	0.97 ± 0.02 ^d^	1.17 ± 0.03 ^c^	1.63 ± 0.06 ^a^	1.63 ± 0.07 ^a^
C10:0	Capric	0.19 ± 0.01 ^g^	2.45 ± 0.06 ^c^	4.37 ± 0.03 ^b^	5.39 ± 0.03 ^a^	2.09 ± 0.06 ^d^	1.00 ± 0.02 ^f^	1.43 ± 0.07 ^e^	1.82 ± 0.21 ^d^
C12:0	Lauric	4.82 ± 0.37 ^c^	7.28 ± 0.01 ^b^	1.69 ± 0.27 ^d^	2.25 ± 0.04 ^d^	5.87 ± 0.14 ^c^	7.46 ± 0.19 ^b^	8.68 ± 0.31 ^a^	5.08 ± 0.09 ^c^
C14:0	Myristic	6.28 ± 0.10 ^b^	8.08 ± 0.05 ^a^	4.28 ± 0.27 ^c^	5.13 ± 0.14 ^b^	7.51 ± 0.43 ^a^	3.09 ± 0.29 ^d^	4.44 ± 0.03 ^c^	3.95 ± 0.60 ^c^
C15:0	Pentadecylic	0.49 ± 0.01 ^b^	0.62 ± 0.01 ^a^	0.45 ± 0.01 ^b^	0.50 ± 0.01 ^b^	0.66 ± 0.00 ^a^	ND	0.04 ± 0.00 ^c^	ND
C16:0	Palmitic	18.30 ± 0.38 ^b^	20.68 ± 0.12 ^b^	22.89 ± 0.55 ^b^	15.63 ± 1.67 ^b^	27.99 ± 0.73 ^a^	25.94 ± 2.70 ^a^	21.81 ± 0.55 ^b^	28.67 ± 2.73 ^a^
C17:0	Margaric	0.33 ± 0.01 ^b^	0.34 ± 0.02 ^b^	0.39 ± 0.01 ^a^	0.39 ± 0.01 ^a^	0.36 ± 0.02 ^b^	0.29 ± 0.03 ^b^	0.27 ± 0.01 ^b^	0.24 ± 0.00 ^c^
C18:0	Stearic	6.11 ± 0.34 ^a^	5.99 ± 0.06 ^a^	6.28 ± 0.06 ^a^	5.46 ± 0.34 ^a^	6.08 ± 0.07 ^a^	3.56 ± 0.14 ^b^	3.90 ± 0.05 ^b^	3.16 ± 0.02 ^c^
C19:0	Nonadecanoic	0.07 ± 0.01 ^b^	0.07 ± 0.01 ^b^	0.10 ± 0.00 ^a^	0.09 ± 0.00 ^a^	0.06 ± 0.01 ^b^	ND	ND	0.01 ± 0.00 ^c^
C20:0	Arachidic	0.21 ± 0.00 ^b^	0.19 ± 0.00 ^b^	0.27 ± 0.02 ^a^	0.25 ± 0.01 ^b^	0.22 ± 0.00 ^b^	0.32 ± 0.00 ^a^	0.31 ± 0.02 ^a^	0.25 ± 0.01 ^b^
C22:0	Behenic	0.30 ± 0.01 ^c^	0.28 ± 0.01 ^c^	0.42 ± 0.00 ^b^	0.42 ± 0.03 ^b^	0.15 ± 0.01 ^e^	0.17 ± 0.00 ^e^	0.24 ± 0.02 ^d^	0.49 ± 0.01 ^a^
C24:0	Lignoceric	0.15 ± 0.00 ^b^	0.12 ± 0.00 ^b^	0.22 ± 0.03 ^a^	0.17 ± 0.00 ^ab^	ND	0.14 ± 0.00 ^b^	ND	0.24 ± 0.01 ^a^
Total SFAs		40.68 ± 2.80 ^b^	50.48 ± 0.02 ^a^	44.9 ± 0.31 ^b^	40.11 ± 1.78 ^b^	55.12 ± 0.26 ^a^	43.14 ± 2.22 ^b^	42.75 ± 0.72 ^b^	45.54 ± 3.50 ^b^
Monounsaturated fatty acids (MUFAs)									
C14:1 *n*5	Myristoleic	0.43 ± 0.02 ^b^	0.59 ± 0.01 ^a^	0.05 ± 0.00 ^c^	0.07 ± 0.01 ^c^	0.62 ± 0.00 ^a^	ND	ND	ND
C16:1 *n*7	Palmitoleic	0.89 ± 0.02 ^a^	1.06 ± 0.00 ^a^	0.51 ± 0.00 ^b^	0.50 ± 0.00 ^b^	1.06 ± 0.03 ^a^	1.20 ± 0.05 ^a^	1.02 ± 0.03 ^a^	1.13 ± 0.11 ^a^
C17:1 *n*9	cis-10-heptadecenoic	0.12 ± 0.01 ^a^	0.12 ± 0.01 ^a^	0.18 ± 0.02 ^a^	0.16 ± 0.01 ^a^	0.14 ± 0.02 ^a^	0.16 ± 0.02 ^a^	0.13 ± 0.00 ^a^	0.05 ± 0.01 ^b^
C18:1 *n*9	Oleic	32.32 ± 1.82 ^b^	29.43 ± 0.32 ^b^	35.58 ± 0.47 ^ab^	41.15 ± 2.04 ^a^	27.14 ± 0.06 ^b^	36.00 ± 1.45 ^ab^	34.84 ± 0.94 ^ab^	33.37 ± 3.48 ^b^
C20:1 *n*9	Eicosenoic	0.27 ± 0.02 ^b^	0.23 ± 0.01 ^b^	0.31 ± 0.01 ^a^	0.29 ± 0.01 ^ab^	0.26 ± 0.01 ^b^	0.30 ± 0.01 ^ab^	0.33 ± 0.03 ^a^	0.32 ± 0.01 ^a^
Total MUFAs		35.71 ± 1.89 ^b^	32.97 ± 0.34 ^b^	38.61 ± 0.45 ^ab^	44.10 ± 2.01 ^a^	30.81 ± 0.06 ^b^	39.36 ± 1.44 ^ab^	37.68 ± 0.99 ^ab^	36.80 ± 3.52 ^b^
Polyunsaturated fatty acids (PUFAs)									
C18:2 *n*6	Linoleic (LA)	14.33 ± 1.50 ^b^	12.57 ± 0.01 ^b^	11.99 ± 0.57 ^b^	11.91 ± 0.52 ^b^	10.23 ± 0.12 ^bc^	14.99 ± 0.57 ^ab^	16.92 ± 0.08 ^a^	14.83 ± 0.17 ^ab^
C18:3 *n*6	γ-linolenic acid (GLA)	0.04 ± 0.00 ^a^	0.03 ± 0.00 ^a^	0.03 ± 0.00 ^a^	0.03 ± 0.00 ^a^	0.03 ± 0.00 ^a^	ND	0.03 ± 0.00 ^a^	0.04 ± 0.00 ^a^
C20:4 *n*6	Arachidonic (AA)	0.48 ± 0.03 ^a^	0.31 ± 0.00 ^b^	0.47 ± 0.07 ^a^	0.42 ± 0.01 ^ab^	0.39 ± 0.01 ^ab^	0.37 ± 0.01 ^b^	0.49 ± 0.02 ^a^	0.59 ± 0.01 ^a^
C18:3 *n*3	α-linolenic (ALA)	1.67 ± 0.00 ^a^	1.43 ± 0.01 ^b^	1.80 ± 0.03 ^a^	1.72 ± 0.03 ^a^	0.96 ± 0.02 ^c^	1.62 ± 0.07 ^a^	1.52 ± 0.11 ^ab^	1.54 ± 0.02 ^ab^
C20:5 *n*3	Eicosapentaenoic (EPA)	0.13 ± 0.00 ^a^	0.14 ± 0.00 ^a^	0.10 ± 0.00 ^b^	0.10 ± 0.00 ^b^	ND	0.10 ± 0.00 ^b^	0.09 ± 0.01 ^b^	ND
C22:6 *n*3	Docosahexaenoic (DHA)	0.47 ± 0.02 ^b^	0.54 ± 0.00 ^a^	0.46 ± 0.03 ^b^	0.41 ± 0.01 ^b^	0.28 ± 0.05 ^c^	0.33 ± 0.01 ^bc^	0.43 ± 0.01 ^b^	0.56 ± 0.03 ^a^
Total PUFAs		17.5 ± 1.43 ^a^	15.38 ± 0.01 ^ab^	15.35 ± 0.65 ^ab^	15.11 ± 0.49 ^b^	12.28 ± 0.11 ^c^	17.47 ± 0.53 ^a^	19.50 ± 0.04 ^a^	17.64 ± 0.25 ^a^
Branched-chain fatty acids (BCFAs)									
*iso*C13:0	Iso-tridecanoic	0.03 ± 0.00 ^b^	0.02 ± 0.03 ^b^	0.02 ± 0.00 ^b^	0.02 ± 0.00 ^b^	0.05 ± 0.00 ^a^	ND	ND	ND
*iso*C14:0	Iso-tetradecanoic	0.03 ± 0.00 ^a^	0.03 ± 0.00 ^a^	0.04 ± 0.00 ^a^	0.05 ± 0.00 ^a^	0.04 ± 0.00 ^a^	ND	ND	ND
*iso*C15:0	Iso-pentadecylic	0.13 ± 0.04 ^a^	0.11 ± 0.01 ^a^	0.09 ± 0.00 ^a^	0.10 ± 0.01 ^a^	0.10 ± 0.01 ^a^	ND	ND	ND
*anteiso*C15:0	Anteiso-pentadecylic	0.21 ± 0.01 ^a^	0.22 ± 0.01 ^a^	0.14 ± 0.00 ^a^	0.17 ± 0.01 ^a^	0.24 ± 0.02 ^a^	ND	ND	ND
*iso*C16:0	Isopalmitic	0.11 ± 0.01 ^a^	0.12 ± 0.01 ^a^	0.12 ± 0.00 ^a^	0.12 ± 0.01 ^a^	0.11 ± 0.00 ^a^	ND	ND	ND
Total BCFAs		0.50 ± 0.04 ^a^	0.50 ± 0.01 ^a^	0.41 ± 0.01 ^a^	0.45 ± 0.02 ^a^	0.54 ± 0.00 ^a^	ND	ND	ND
Trans fatty acids (TFAs)									
C16:1 *t*6	Palmitelaidic	0.17 ± 0.00 ^a^	0.17 ± 0.01 ^a^	0.20 ± 0.02 ^a^	0.17 ± 0.00 ^ab^	0.15 ± 0.01 ^a^	0.02 ± 0.00 ^b^	0.01 ± 0.00 ^b^	ND
C18:1 *t*6	Petroselaidic	0.11 ± 0.01 ^a^	0.09 ± 0.00 ^b^	0.09 ± 0.00 ^b^	0.06 ± 0.00 ^c^	0.10 ± 0.00 ^ab^	ND	ND	ND
C18:1 *t*9	Elaidic	0.07 ± 0.00 ^a^	0.08 ± 0.00 ^a^	ND	0.08 ± 0.00 ^a^	0.08 ± 0.01 ^a^	ND	ND	ND
C18:1 *t*11	Vaccenic (VA)	1.33 ± 0.08 ^a^	1.20 ± 0.01 ^a^	1.69 ± 0.01 ^a^	1.62 ± 0.00 ^a^	1.26 ± 0.01 ^a^	1.68 ± 0.08 ^a^	1.35 ± 0.12 ^a^	1.93 ± 0.05 ^a^
C18:2 *c*9*t*11	Rumenic (RA, CLA)	0.21 ± 0.02 ^b^	0.19 ± 0.01 ^b^	0.31 ± 0.00 ^a^	0.30 ± 0.01 ^a^	0.21 ± 0.01 ^b^	ND	ND	ND
C18:2 *t*9*t*12	Trans isomers of linoleic acid	0.09 ± 0.00 ^a^	0.10 ± 0.00 ^a^	0.11 ± 0.01 ^a^	0.12 ± 0.01 ^a^	0.10 ± 0.01 ^a^	0.03 ± 0.00 ^b^	ND	0.05 ± 0.00 ^b^
C18:2 *t*11*t*15		0.08 ± 0.01 ^a^	0.07 ± 0.00 ^a^	0.08 ± 0.00 ^a^	0.10 ± 0.00 ^a^	0.08 ± 0.01 ^a^	0.03 ± 0.00 ^b^	0.02 ± 0.00 ^b^	0.03 ± 0.00 ^b^
Total TFAs		2.06 ± 0.06 ^b^	1.99 ± 0.02 ^b^	2.48 ± 0.03 ^a^	2.44 ± 0.02 ^a^	1.98 ± 0.02 ^b^	1.76 ± 0.08 ^b^	1.38 ± 0.12 ^c^	2.01 ± 0.06 ^b^
Fatty acid ratios									
PUFA *n*6/PUFA *n*3		6.71 ± 0.69 ^ab^	6.29 ± 0.04 ^ab^	5.50 ± 0.27 ^c^	5.78 ± 0.35 ^b^	8.90 ± 0.29 ^a^	7.52 ± 0.49 ^a^	8.56 ± 0.61 ^a^	7.40 ± 0.10 ^a^
DHA:LA		0.03:1	0.04:1	0.04:1	0.03:1	0.03:1	0.02:1	0.03:1	0.04:1
DHA:AA		0.98:1	1.74:1	0.98:1	0.98:1	0.72:1	0.89:1	0.88:1	0.95:1
LA:ALA		8.58:1	8.79:1	6.66:1	6.92:1	10.66:1	9.25:1	11.13:1	9.63:1

Abbreviations: IF—infant formula, SFAs—saturated fatty acids, MUFAs—monounsaturated fatty acids, PUFAs—polyunsaturated fatty acids, TFAs—trans fatty acids, BCFAs—branched chain fatty acids, ND—not detected. Mean values in lines marked with different letters (a–g) are significantly different at *p* < 0.05.

**Table 3:** Assessment of compliance of total PUFA and individual PUFA contents in IFs, %.

IFs	PUFA		LA		ALA		AA		DHA		EPA	
	VL	VM	VL	VM	VL	VM	VL	VM	VL	VM	VL	VM
K-CM1	15.63	17.50	14.00	14.33	1.72	1.67	0.52	0.48	0.52	0.47	0.11	0.13
K-CM2	16.13	15.38	13.71	12.57	1.68	1.43	0.29	0.31	0.55	0.54	0.12	0.14
K-GM1	15.92	15.35	12.01	11.88	1.59	1.80	0.48	0.47	0.48	0.46	ND	-
K-GM2	15.92	15.11	12.01	11.91	1.59	1.72	0.48	0.42	0.45	0.41	ND	-
S-PH	11.76	12.28	10.91	10.23	1.09	0.96	0.20	**0.39**	0.44	**0.28**	ND	-
S-S	18.18	17.47	14.85	14.99	1.66	1.62	ND	-	0.48	**0.33**	ND	-
S-A	16.18	17.64	13.09	14.83	1.32	1.54	0.50	0.59	0.50	0.56	ND	-

Abbreviations: IF—infant formula, PUFAs—polyunsaturated fatty acids, LA—linoleic acid, ALA—α—linolenic acid, AA—arachidonic acid, DHA—docosahexaenoic acid, EPA—eicosapentaenoic acid, VL—value labeled—% of fatty acids declared by the manufacturer of IFs, VM—value marked—% of fatty acids determined in analytical studies, ND—no data. Values in bold indicate differences of 20% or more in the determined fatty acid concentrations from those declared by the manufacturer. Declared values on the labels were considered as 100% for both the total PUFA content and individual PUFAs.

**Table 4:** The lipid quality indices estimated for the tested conventional and specialist IFs.

Lipid Quality Indices	K-CM1	K-CM2	K-GM1	K-GM2	S-PH	S-S	S-FH	S-A
DFA	59.32 ^a^	54.34 ^b^	60.24 ^a^	64.67 ^a^	49.17 ^c^	60.39 ^a^	61.08 ^a^	57.60 ^b^
OFA	29.4 ^bc^	36.04 ^b^	28.86 ^bc^	23.01 ^c^	41.37 ^a^	36.49 ^b^	34.93 ^b^	37.70 ^b^
AI	0.91 ^b^	1.25 ^ab^	0.77 ^c^	0.65 ^c^	1.48 ^a^	0.81 ^bc^	0.84 ^bc^	0.91 ^b^
TI	0.95 ^b^	1.17 ^b^	1.01 ^b^	0.74 ^c^	1.68 ^a^	0.97 ^b^	0.89 ^bc^	1.10 ^b^
H/H	1.64 ^b^	1.21 ^b^	1.71 ^b^	2.38 ^a^	0.93 ^b^	1.44 ^b^	1.53 ^b^	1.32 ^b^

Abbreviations: DFAs—index of desirable fatty acids, OFAs—index of hypercholesterolemic fatty acids, AI—index of atherogenicity, TI—index of thrombogenicity, H/H hypocholesterolemic/hypercholesterolemic ratio. Mean values in lines marked with different letters (a–c) are significantly different at *p* < 0.05.

## Data Availability

The data presented in this study is available in the article and Supplementary Materials.

## Supplementary Materials

The following supporting information can be downloaded at: https://www.mdpi.com/article/10.3390/molecules30153221/s1, Figure S1: Chromatograms of separations obtained from a used standard (A), and of a sample of infant formula (B).

## Abbreviations

The following abbreviations are used in this manuscript:

AA	Arachidonic acid
AI	Index of atherogenicity
ALA	α-Linolenic
BCFAs	Branched-chain fatty acids
CLA	Conjugated linoleic acid
DFAs	Index of desirable fatty acids
DHA	Docosahexaenoic fatty acid
EPA	Eicosaenoic fatty acid
HM	Human milk
IF	Infant formula
HCA	Hierarchical cluster analysis
H/H	Hypocholesterolemic/Hypercholesterolemic ratio
LA	Linoleic acid
LCPUFAs	Long-chain polyunsaturated fatty acids
MCFAs	Medium-chain fatty acids
MUFAs	Monounsaturated fatty acids
OFAs	Index of hypercholesterolemic fatty acids
PUFAs	Polyunsaturated fatty acids
SCFAs	Short-chain fatty acids
SFAs	Saturated fatty acids
TFA	Trans fatty acid
TI	Index of thrombogenicity
VA	Vaccenic acid
