# Supplementary material for: Comparative Evaluation of Fat Quality in Conventional and Specialist Infant Formulas
Source: Molecules. 2025 Jul 31;30(15):3221. doi: 10.3390/molecules30153221 (PMC12348159; doi:10.3390/molecules30153221)
Supplement: Supplementary file 1 [file molecules-30-03221-s001.zip › molecules-3761137-supplementary.pdf]

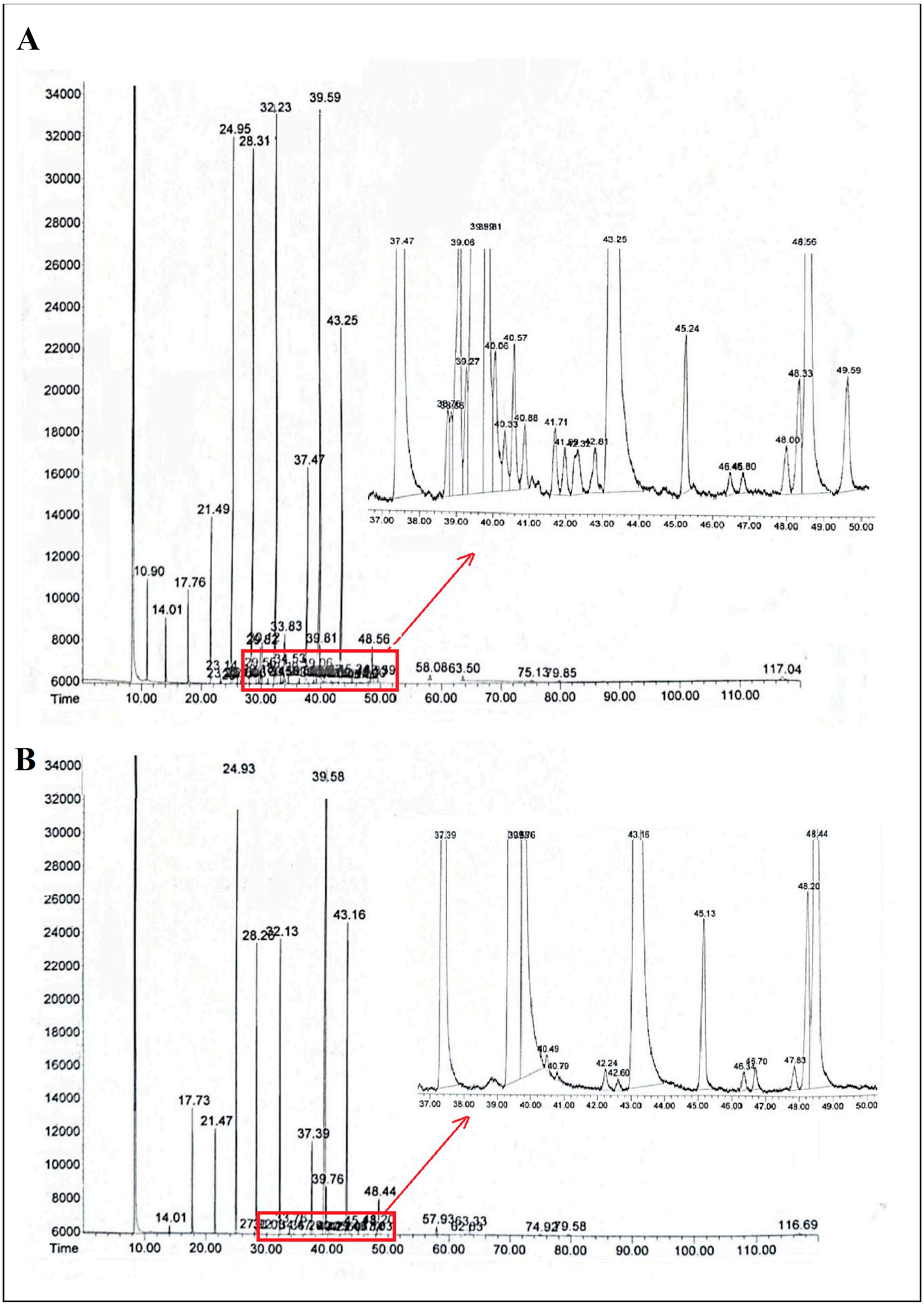

Figure S1. Chromatograms of separations obtained from a used standard (A), and of a sample of infant formula (B).
